# Editorial: Forebrain control of breathing and sudden death in epilepsy

**DOI:** 10.3389/fncir.2023.1212172

**Published:** 2023-05-23

**Authors:** Steven A. Crone, Brian J. Dlouhy, Christina Gross, Russell S. Ray

**Affiliations:** ^1^Division of Pediatric Neurosurgery, Cincinnati Children's Hospital Medical Center, Cincinnati, OH, United States; ^2^Division of Developmental Biology, Cincinnati Children's Hospital Medical Center, Cincinnati, OH, United States; ^3^Department of Neurosurgery, University of Cincinnati College of Medicine, Cincinnati, OH, United States; ^4^Department of Neurosurgery, University of Iowa, Iowa City, IA, United States; ^5^Iowa Neuroscience Institute, University of Iowa, Iowa City, IA, United States; ^6^Pappajohn Biomedical Institute, University of Iowa, Iowa City, IA, United States; ^7^Division of Neurology, Cincinnati Children's Hospital Medical Center, Cincinnati, OH, United States; ^8^Department of Pediatrics, University of Cincinnati College of Medicine, Cincinnati, OH, United States; ^9^Memory Brain Research Center, Baylor College of Medicine, Houston, TX, United States; ^10^Department of Neuroscience, Baylor College of Medicine, Houston, TX, United States; ^11^McNair Medical Institute, Houston, TX, United States

**Keywords:** forebrain, control of breathing, sudden death in epilepsy, SUDEP, epilepsy, respiration, seizure

Sudden Unexpected Death in Epilepsy (SUDEP) is the unexpected death of a person with epilepsy who was otherwise healthy and no other cause of death was found (Nashef et al., 2012). SUDEP is the second leading neurological cause of lost years of life, behind stroke, and is the leading cause of death among persons with drug-resistant epilepsy (Thurman et al., 2014). Both clinical and basic research studies have implicated respiratory deficits as a major contributor to SUDEP (Devinsky et al., 2016). For example, a study of patients who died while in epilepsy monitoring units found that all SUDEP cases exhibited post-seizure cardiorespiratory abnormalities, with terminal apneas preceding terminal asystole (Ryvlin et al., 2013). However, our current understanding of the causes of respiratory abnormalities in persons with epilepsy that may lead to SUDEP is incomplete.

The forebrain is the most common site of seizures and contributes to the regulation of breathing through connections to brainstem respiratory regions and/or spinal circuits that control respiratory motor neurons. Schottelkotte and Crone review the forebrain regions that have been implicated in the control of breathing. They describe functional evidence of a role in breathing for forebrain structures that include the cerebral cortex, hippocampus, amygdala, thalamus, and hypothalamus. A better understanding of the role of each forebrain region in the control of breathing may improve our understanding of how respiratory failure might occur in cases of SUDEP.

SUDEP is thought to occur primarily during sleep (Buchanan et al., 2021). Joyal et al. review the relationships between sleep state/time of day, seizures, and respiratory function and how they could contribute independently to the risk of SUDEP. Other factors, such as neurotransmitter levels, airway obstruction and arousal responses, may also contribute. It is important to note that increased monitoring during sleep using devices or having another person sleep in the same room can decrease SUDEP risk (Harden et al., 2017).

Several hypotheses have been proposed to explain how epilepsy could disrupt the control of breathing. Mulkey and Milla review potential mechanisms by which seizure activity in the forebrain could propagate to the brainstem to affect breathing. They describe how seizures could activate descending inhibitory drive to brainstem respiratory nuclei or how spreading depolarizations within the brainstem could impair breathing. A critical role for astrocytes as well as epilepsy related mutations in this process is discussed. Bauer et al. review *in vivo* and *ex vivo* techniques to further study the potential mechanisms of SUDEP in animal models. They describe how synaptic or non-synaptic (i.e., spreading depolarizations) mechanisms could disrupt brainstem regions important for breathing and/or cardiac function in epilepsy. Original research by Wenker et al. refute the hypothesis that cortical neurons directly drive apnea in the Scn8a mouse model of epilepsy. They found that silencing forebrain neurons could reduce seizure threshold but did not prevent apneas during the tonic phase of seizures. They propose that brainstem circuitry most likely drives tonic phase apneas that are thought to contribute to SUDEP. Different mechanisms (or combinations of risk factors) may be engaged in different people, necessitating additional research using a variety of approaches and animal models.

There are currently no established biomarkers to identify individuals that are vulnerable to SUDEP. Potential predictors of SUDEP under investigation include impaired chemosensory responses, depressed arousal/resuscitation responses, altered brain activity, abnormal respiratory patterns, or altered neurotransmitter release. For example, prior studies have shown that some epilepsy patients have a decreased ability to respond to increased levels of carbon dioxide in the blood, which correlates with longer episodes of respiratory depression following a seizure (Sainju et al., 2019). Altered chemosensory reflexes could impair auto-resuscitation following a seizure, increasing SUDEP risk. Hampson et al. use functional magnetic resonance imaging (fMRI) to examine changes in brain activity during increased CO_2_ exposure in persons with epilepsy compared to healthy controls. They show enhanced activation of the dorsal raphe (which contains CO_2_ sensitive serotonergic neurons) as well as altered functional connectivity between the brainstem and adjacent subcortical areas in persons with epilepsy. These results suggest that the respiratory system may be less able to handle respiratory challenges in persons with epilepsy. Ciumas et al. review the current methods to use fMRI to investigate central respiratory control in human patients. The authors discuss the challenges associated with studying the brainstem nuclei involved in breathing and summarize the various methods that can be used to overcome these challenges. They review examples of studies that have used fMRI to study breathing in various conditions, including epilepsy. Functional imaging is a promising tool that may be useful for identifying individuals that are most at risk for SUDEP as well as probing the mechanisms leading to SUDEP.

There are currently no therapeutic approaches to prevent SUDEP and research in this area presents many challenges, including difficulty assessing which patients are at risk, insufficient understanding of the underlying mechanisms, a lack of animal models to recapitulate all aspects of SUDEP, and the diversity of patients at risk for SUDEP. Bauer et al. and Mulkey and Milla describe the current state of research aiming to prevent cardiorespiratory abnormalities that could lead to SUDEP. These include methods to stimulate (or inhibit) specific brain regions implicated in SUDEP as well as methods to prevent initiation or propagation of spreading depolarizations. Two original research articles describe potential novel treatment targets: Collard et al. work indicates that the neurotransmitter Galanin holds promise as a potential future treatment because either systemic or central nervous system delivery of galanin analogs could prevent seizure induced respiratory arrest in mouse electroshock seizure models. Fukushi et al. show that inhibition of microglia activation with minocycline delayed seizures induced by hypoxia and reduced the incidence of post-seizure respiratory arrest.

Epilepsy patients may undergo a vicious cycle whereby seizures impair respiratory responses and impaired breathing increases the incidence or severity of seizures. The complicated interactions between epilepsy and breathing control indicate that collaborations between researchers and clinicians investigating epilepsy and experts on the control of breathing could advance our understanding of SUDEP and accelerate prevention.

## Author contributions

All authors listed have made a substantial, direct, and intellectual contribution to the work and approved it for publication.
